# Comment on “The optimal timing of post-treatment sampling for the assessment of anthelminthic drug efficacy against *Ascaris* infections in humans”

**DOI:** 10.1016/j.ijpddr.2018.05.004

**Published:** 2018-05-18

**Authors:** Jürgen Krücken, Kira Fraundorfer, Jean Claude Mugisha, Sabrina Ramünke, Kevin C. Sifft, Dominik Geus, Felix Habarugira, Jules Ndoli, Augustin Sendegeya, Caritas Mukampunga, Toni Aebischer, Janina McKay-Demeler, Jean Bosco Gahutu, Frank P. Mockenhaupt, Georg von Samson-Himmelstjerna

**Affiliations:** a Institute for Parasitology and Tropical Veterinary Medicine, Freie Universität Berlin, Robert-von-Ostertag-Str. 7-13, 14163 Berlin, Germany; b University Teaching Hospital of Butare, University of Rwanda, Butare, Rwanda; c Institute of Tropical Medicine and International Health, Charité-University Medicine Berlin, Augustenburger Platz 1, 13353 Berlin, Germany; d Mycotic and Parasitic Agents and Mycobacteria, Department of Infectious Diseases, Robert Koch-Institute, Berlin, Germany; e Dawbuts, 9 Mitchell St, Camden, NSW, 2750 Australia

## Abstract

A recent publication by Levecke et al. (Int. J. Parasitol, 2018, 8, 67–69) provides important insights into the kinetics of worm expulsion from humans following treatment with albendazole. This is an important aspect of determining the optimal time-point for post treatment sampling to examine anthelmintic drug efficacy. The authors conclude that for the determination of drug efficacy against Ascaris, samples should be taken not before day 14 and recommend a period between days 14 and 21. Using this recommendation, they conclude that previous data (Krücken et al., 2017; Int. J. Parasitol, 7, 262–271) showing a reduction of egg shedding by 75.4% in schoolchildren in Rwanda and our conclusions from these data should be interpreted with caution. In reply to this, we would like to indicate that the very low efficacy of 0% in one school and 52–56% in three other schools, while the drug was fully efficient in other schools, cannot simply be explained by the time point of sampling. Moreover, there was no correlation between the sampling day and albendazole efficacy. We would also like to indicate that we very carefully interpreted our data and, for example, nowhere claimed that we found anthelmintic resistance. Rather, we stated that our data indicated that benzimidazole resistance may be suspected in the study population. We strongly agree that the data presented by Levecke et al. suggests that recommendations for efficacy testing of anthelmintic drugs should be revised.

## Graphical abstract

**Figure:**
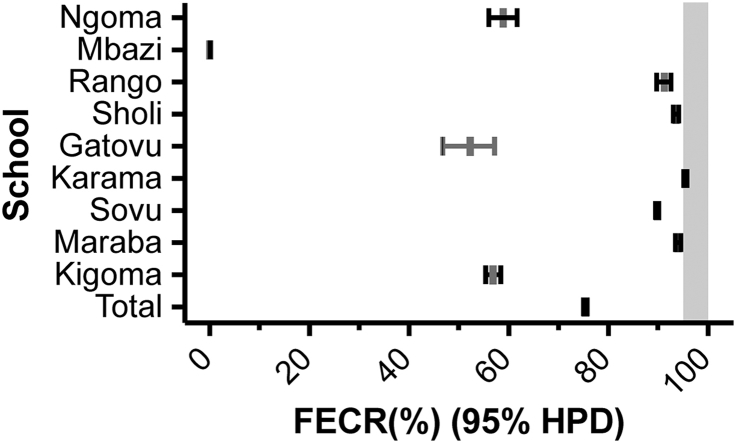

A recent publication by Levecke et al. (2018) provided interesting information on the kinetics of expulsion of *Ascaris lumbricoides* in humans following benzimidazole treatment. They show that expulsion of *Ascaris* worms can occur as late as 10 days post treatment with 400 mg albendazole and that this late worm expulsion affects faecal egg count reduction (FECR = egg reduction rate, ERR), which was only 89.4% on day 7 post treatment in their dataset but reached 100% at day 10 post treatment. These observations add to the ongoing discussion on how to best monitor the efficacy of anthelmintic chemotherapy.

Monitoring anthelmintic efficacy in humans currently relies solely on faecal egg counts. Egg counts, however, suffer from several confounding factors such as the non-homogenous distribution of eggs in faeces, among others (Torgerson et al., 2014; Levecke et al., 2015; Wang et al., 2017). Novel statistical methods partially solve these issues and account for effects of variation on confidence intervals. Still, only few data sets are available on egg counts following treatment. Of note, the eggCounts package (Torgerson et al., 2014; Wang et al., 2017) developed by Reinhard Furrer's group requires such data for optimization and for informing respective Bayesian models to calculate FECRs and their 95% confidence intervals.

The optimal timing of post-treatment sample collection depends on various factors including host/parasite species, drug and treatment regimen. For instance, in the veterinary field, Coles et al. (2006) recommended stool sampling 3–7 days post-treatment for levamisole, 8–10 days post-treatment for the benzimidazoles and 14–17 days post-treatment for the macrocyclic lactones.

In a recent study among Rwandan schoolchildren (Krücken et al., 2017), we collected FEC data on day 7–10 post treatment with albendazole. We found a very high variation in the effect of treatment based on FECR determinations between children attending different schools in a district in southern Rwanda, and raised the suspicion that FECRs <95% could indicate reduced efficacy, i.e. emerging benzimidazole resistance. This interpretation is now criticized by Levecke and colleagues based on their findings that a FECR of 89% on day 7 can still reach 100% on days 14 and 21 (Levecke et al. (2018)). We fully agree with Levecke and colleagues that future study designs should take this delayed clearance in humans into account. We also support the conclusion that recommendations for gathering FECR data should be revised and that the suggestions made by Vercruysse et al. (2011) suggesting a 7–21 days interval as well as the WHO guidelines should be openly discussed and presumably reformed. Optimally, this should involve experts from different working groups and include also researchers interested in other parasites shed with the faeces such as schistosomes or *Giardia duodenalis* to allow the broadest and most precise collection of data possible from such samples.

However, the large differences in FECR noted in our study (Krücken et al., 2017) cannot be explained by a potentially suboptimal time of sampling on days 7–10. Notably, we observed a FECR of 0% in one school and FECRs in further three schools in the range 52–56%. In contrast, other schools had FECRs higher than 90% or even 95%. This variation has not been equally considered and weighted by Levecke et al. (2018) with respect to the overall conclusion of suspected emerging BZ resistance (Krücken et al., 2017). While the school showing 0% FECR was indeed revisited on day 7 post treatment, the three schools with 52–56% FECR were all resampled on day 10. In contrast, at the two schools with the highest FECR of 94–95% post treatment samples were collected on day 7. This clearly indicates that the sampling time cannot be the main factor explaining low FECRs in our data set.

We are only beginning to understand how studies on FECR should best be conducted and interpreted in the case of human infections. In the veterinary field, there is plenty of data available showing that interpretation of FECRs require host-specific modifications. Studies such as ours and that by Levecke et al. (2018) are important to evaluate how criteria applied in veterinary medicine should be adapted for human infections. The logistic challenges to obtain such data are considerable including the daily collection of complete faecal matter over two weeks and the quantification of worms in these samples under field conditions. However, such efforts will be rewarded by well-founded criteria allowing us to answer the burning question: Is anthelimintic resistance emerging and putting mass deworming efforts in jeopardy?

We believe that our own data (Krücken et al., 20187) and that of Levecke et al. (2018) highlight the need and ways to progress towards this goal, respectively.
